# Bovine myoblast cell production in a microcarriers-based system

**DOI:** 10.1007/s10616-017-0101-8

**Published:** 2017-05-03

**Authors:** Sanne Verbruggen, Daan Luining, Anon van Essen, Mark J. Post

**Affiliations:** Department of Physiology, Maastricht University, Universiteitssingel 50, 6229 ER Maastricht, The Netherlands

**Keywords:** Cell culture, Bioreactor, Microcarriers, Myoblast

## Abstract

For several tissue engineering applications, in particular food products, scaling up culture of mammalian cells is a necessary task. The prevailing method for large scale cell culture is the stirred tank bioreactor where anchor dependent cells are grown on microcarriers suspended in medium. We use a spinner flask system with cells grown on microcarriers to optimize the growth of bovine myoblasts. Freshly isolated primary cells were seeded on microcarriers (Synthemax^®^, CellBIND^®^ and Cytodex^®^ 1 MCs). In this study, we provide proof of principle that bovine myoblasts can be cultured on microcarriers. No major differences were observed between the three tested microcarriers, except that sparsely populated beads were more common with CellBIND^®^ and Synthemax^®^ II beads suggesting a slower initiation of exponential growth than on Cytodex^®^. We also provide direct evidence that bovine myoblasts display bead-to-bead transfer. A remarkable pick up of growth was observed by adding new MCs. Bovine myoblasts seem to behave like human mesenchymal stem cells. Thus, our results provide valuable data to further develop and scale-up the production of bovine myoblasts as a prerequisite for efficient and cost-effective development of cultured meat. Applicability to other anchorage dependent cells can extend the importance of these results to cell culture for medical tissue engineering or cell therapy.

## Introduction

Large scale culture of mammalian, anchor dependent cells is a necessary condition for cell therapy and tissue engineering. We recently started to use cell and tissue culture for food application where the required scale is even orders of magnitudes higher. Large scale cell production is not only necessary to achieve large numbers of cells but also to reach an efficiency in number of cells grown per unit of medium, leading to resource efficiency. Resource efficiency and cost-effectiveness of cell culture is much more important in food production than in the medical industry (Van Der Weele and Tramper 2014; Post and Van Der Weele 2014). Of the scalable cell culture systems, the stirred tank bioreactor with cells grown on microcarriers is most commonly used (Nienow 2006). One of the advantages is that microcarriers can provide a larger surface area per unit volume of medium compared to a tissue culture flask (Nienow 2006). The smaller laboratory scale stirrer flask serves as a model for the stirred tank bioreactor.

One of the variables that needs to be optimized in stirred flask cell culture is the choice of microcarrier as mammalian cells are typically anchor-dependent. Four groups of microcarriers are being distinguished based on charge, coating, surface, and size.

Cytodex^®^ 1 is a positively charged, non-porous polystyrene microcarrier that is frequently used to culture mesenchymal stem cells in stirred tank bioreactors (Chen et al. 2013; Frauenschuh et al. 2007; Schop et al. 2008). Others are negatively charged and resemble the properties of 2D cell culture plates. The second group of microcarriers is coated with collagen (Cytodex^®^ 3) or synthetic ECM components (Synthemax^®^ II) designated for cells with low adhesive capacity and for ease of cell harvesting. Myoblasts do not adhere well to uncoated plastic and are therefore commonly cultured on flasks or dishes coated with a mixture of collagen and Matrigel™ (Macfelda et al. 2007; Stern et al. 2009). We hypothesized that microcarrier-based culture methods must be optimized for myoblasts separately from the already established methods for mesenchymal stem cells and iPS cells.

Cytodex^®^ 3 has been used to culture mesenchymal stem cells (Hewitt et al. 2011) and induced pluripotent (iPS) (Gupta et al. 2016) cells. To increase the adhesive surface and to offer better protection against shear stress in the stirred tank bioreactor, microcarriers have been made macro porous with pore sizes ranging from 10 to 70 µm. The Cultispher^®^ is most commonly used for mesenchymal stem cell expansion (Eibes et al. 2010; Ferrari et al. 2014; Wu et al. 2003). Agitation and the resultant shear stress is particularly important as too much agitation and resultant shear stress may lead to loss of cells (Croughan and Wang 1989; Stathopoulos and Hellums 1985). However, even during the attachment phase, a certain level of agitation is required to mix the cells, to prevent cell aggregation and to create a homogeneous culture (Nienow 2014). Mesenchymal cells on microcarriers subjected to shear stress in a spinner flask were shown to survive and remain undamaged up to 7 days (Ikonomou et al. 2004).

A recurrent issue in microcarrier-based culture technology is the harvesting of cells from the beads. Conflicting reports on the efficiency of trypsinization exist (Goh et al. 2013) and additional sieving procedures have been used to optimize cell retrieval from the microcarriers (Caruso et al. 2014; Goh et al. 2013). None of these studies pertain to myoblasts.

In this report, we describe our experience with Cytodex^®^ 1, CellBIND^®^ and Synthemax^®^ in a stirrer flask culture system. Other aspects such as optimal seeding density, bead-to-bead transfer and harvesting efficiency were investigated, with the goal to develop an optimized system for bovine myoblast culture as a source to create meat.

## Materials and methods

### Cell isolation

Bovine myoblasts were isolated from fresh beef from a local slaughter-house (Kusters, Margraten, The Netherlands). In this article we used 3 different donors. Briefly, a piece of muscle was cut in small pieces, and suspended in DMEM with 1% penicilline/streptomycin/amphotericin (P/S/A) and 400 unit/ml collagenase 2 (Worthington, Lakewood, NJ, USA). The tissue was further dissociated with the Gentlemac Dissociater (Miltenyi Biotec, Leiden, Netherlands) at the “Heart_01” program and incubated for 45 min at 37 °C. The suspension was spun down for 10 min at 300 g and the supernatant cultured in a Matrigel™-coated (1:200, BD Bioscience, Breda, Netherlands) cell culture flask.

Growth medium consisted of advanced Dulbecco’s modified Eagle’s medium (adv DMEM, Gibco/Thermo Fisher Scientific), fetal bovine serum (20%, Gibco, Thermo Fisher Scientific), horse serum (10%, Gibco, Thermo Fisher Scientific), penicilline/streptomycin/amphotericin (1%, Gibco, Thermo Fisher Scientific) and 4 mM l-glutamine (Lonza, Basel, Switzerland). Medium was changed twice a week. The myoblasts between passage 2 and 5 were transferred to spinner flasks.

Cells were passaged by rinsing with phosphate-buffered saline (PBS; Mg^2+^ and Ca^2+^ free, Gibco, Thermo Fisher Scientific). Preheated 0,05% Trypsin/EDTA (Gibco, Thermo Fisher Scientific) was added to the cells for 7 min at 37 °C. For the differentiation experiments, the cells were incubated with differentiation medium consisting of adv DMEM, horse serum (2%) and penicillin/streptomycin/amphotericin (1%) and 4 mM l-glutamine.

### Preparation of microcarriers

Microcarriers (Table 2) were prepared according to the manufacturers’ instructions. Briefly, Synthemax^®^ and CellBIND^®^ MCs (sterile MCs) were washed twice with sterile water and Cytodex^®^ 1 was washed with Ca^2+^/Mg^2+^-free phosphate-buffered saline. The Cytodex^®^1 beads were autoclaved at 121 °C for 20 min. Before use, MCs were rinsed in growth medium.

### Cultivation of myoblasts in spinner flask

Microcarriers (5 ml, 100 mg/ml) were added to a 250 ml spinner flask (Corning, Wiesbaden Germany) containing 45 ml growth medium. Cells were added at the indicated seeding densities. The first 24 h, the cells underwent an intermittent stirring (30 min rest, 3 min stirring) regime at 37 °C and 5% CO_2_. Thereafter, 50 ml growth medium was added and the agitation rate was 50 rpm.

### Proliferation assay 2D

Cultured cells were washed two times with PBS and then a small amount of the cellTiter96 AQ_ueous_ reagent (Promega, Leiden, Netherlands) was added to the wells. The reagent was allowed to incubate for 2 h before recording the absorbance at 490 nm with a 96-wells plate reader (Perkin Elmer Victor 3, Waltham, MA, USA).

### Proliferation assay 3D

In the microcarrier experiments, cell number was measured by the Qubit dsDNA BR assay (Invitrogen, Thermo Fisher Scientific) according to the manufacturer’s instructions. Briefly, 1 ml of microcarrier/cell suspension sample was washed with phosphate-buffered saline (PBS; Mg^2+^ and Ca^2+^ free, Gibco, Thermo Fisher Scientific) and dissolved in RLT lysis buffer (Qiagen GmbH, Venlo, Netherlands). The samples were incubated for 15 min at 55 °C in an orbital shaker at 100 rpm. The samples were spun down and the supernatant was added to the Qubit working solution, incubated for 2 min and measured with the Qubit 2.0 Fluorometer (Gibco/Thermo Fisher Scientific). The number of cells measured by Qubit equates to 1.5 × 10^5^ × QF cells/ml, where QF is the fluorescence value supplied by the Qubit fluorometer and assuming 6.6 pg DNA/cell.

### Imaging the cells and microcarriers

The cells were stained with Hoechst 33342 (1 mg/ml, Thermo Fisher Scientific) and incubated for 5 min. The microcarrier/cell suspension was washed twice with phosphate-buffered saline and then dissolved in Optimem I (Thermo Fisher Scientific) The pictures were taken from a plate using fluorescence microscopy (Nikon, Amsterdam, Netherlands). For the bead-to-bead transfer experiments, the freshly added Synthemax^®^ beads were stained with Rhodamine (500 µM, Sigma Aldrich, Zwijndrecht, Netherlands) and incubated for 25 min at RT. The beads were washed three times with phosphate-buffered saline. The labeled beads were then added to the existing culture and the entire culture was subjected to intermittent stirring (30 min rest, 3 min stirring) at 37 °C, 5%CO_2_ overnight.

### Gene expression

Expression analysis was performed on samples taken from the microcarrier/cell suspension; RNA was collected using the RNeasy micro-kit Qiagen with DNAse treatment (Qiagen GmbH). RNA concentration was determined with the NanoDrop microspectrophotometer (Thermo Fisher Scientific). A total of 100 ng RNA per sample was subjected to reverse transcription with IScript cDNA synthese kit (Biorad, Veenendaal, Netherlands). Quantitative polymerase chain reaction (QPCR) was performed by iQ SYBR Green supermix (Biorad) and a primer concentration of 10 mM. Quantitative PCR reactions were run on the CFX Real-timePCR detection system (Biorad). Primers were designed with Mfold (www.idtdna.com/scitools/Application/mfold/) and were synthesized by Eurogentec (Liege, Belgium). Primer sequences are provided in Table 1. Samples were normalized for input based on both β-actin and GAPDH values.

**Table 1:** Primer sequences used for Q-PCR

Gene	Fwd sequence	Rev sequence
GAPDH	TCC-CAA-CGT-GTC-TGT–TGT-GGA-TCT	TGT-TGA-AGT-CGC-AGG-AGA-CAA-CCT
β Actin	GGC-ACC-CAG-CAC-AAT-GAA-GAT-CAA	ATC-GTA-CTC-CTG-CTT-GCT-GAT-CCA
MyoD	TAG-GAG-AGG-CGA-AGG-AAC-TGT-TGT	TCT-GGC-CCA-CGG-AGT-AAC-ATC-AAA
Myogenin	AGC-CTC-CAA-ATC-CAC-TCC-CTG-AAA	AGC-CAC-TGG-CAT-AGG-AAG-AGA-TGA

### Statistical analysis

All data are presented as average and standard error, typically with n = 3 (3 stirrer flasks) per experimental setting. Statistical analyses on cell numbers were performed by ANOVA with a Tukey posthoc analysis, based on the assumption of a normal distribution. Cell numbers in the bead-to-bead transfer experiment were analyzed with a two-way ANOVA with time and protocol as variables and a Tukey posthoc analysis. *P* values smaller than 0.05 were accepted as indication of significant difference. The analyses were performed with GraphPad Prism 7, (GraphPad Software Inc, La Jolla, CA, USA).

## Results

From the wide variety of available microcarriers we made the following selection: Cytodex^®^ 1 (positive charge), Synthemax^®^ II (ECM Coated MC) and CellBIND^®^ (negatively charged MC) (see Table 2), based on binding principles and the desire to avoid additional coating.

**Table 2:** Microcarriers and their features

Microcarrier	Shape	Dimension (μm)	Material	Surface properties	Manufacturer
Cytodex 1	Spherical	190	Cross-linked dextran	Positive charge Hydrophilic DEAE exchanger	GE healthcare
Synthemax II	Spherical	125–212	Polystrene	Synthemax	Corning
Cellbind	Spherical	125–212	Polystrene	Negative charge surface (TC-treated)	Corning

**Fig. 1:**
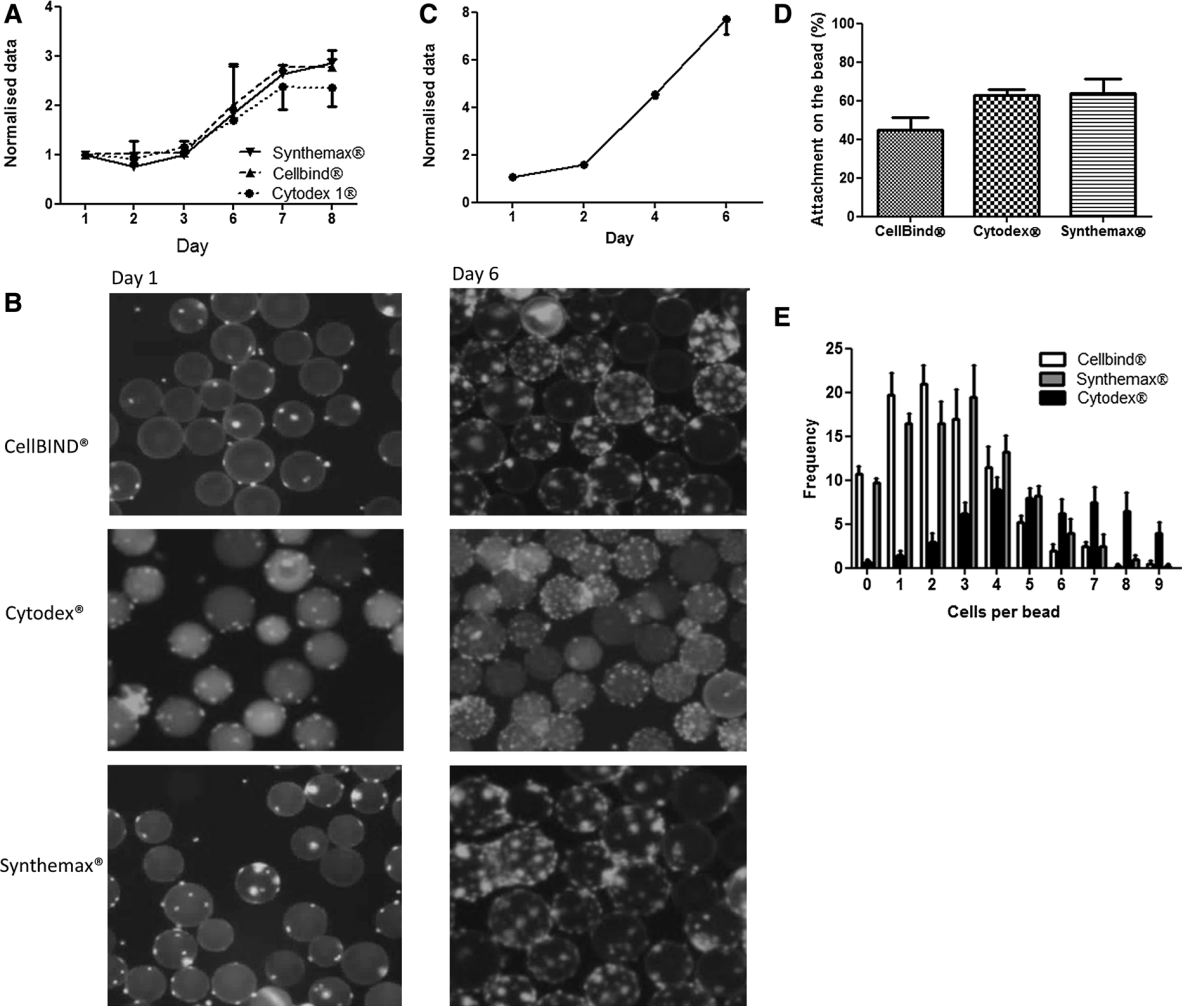
Myoblasts seeded on Cytodex^®^ 1, Synthemax^®^ II and CellBIND^®^ microcarriers using growth medium; seeded at a density of 1 × 10^6^ cells/ml. **a** The *growth curve* of cells for the three microcarriers. DNA (μg/ml) was measured and normalised to the value at day 1 (n = 3 for each day). **b** Photomicrographs of the cell-laden microcarriers at day 1 and day 6. The cells were stained with Hoechst and appear as fluorescent dots (*white* in B&W). **c** 2D proliferation of myoblasts. **d** Attachment of cells to the microcarriers at 24 h expressed as percentage of the total amount of cells added (n = 3 for each type of microcarrier). **e** The amount of cells per bead after 24 h (n = 3)

To test myoblast growth on microcarriers, spinner flasks were seeded at a cell density of 1 × 10^6^ cells/ml. The first 24 h we used an intermittent stirring regime in order to allow efficient cell distribution and attachment. The growth kinetics for all microcarrier-based cultures exhibited a lag phase of 2 days before an exponential phase was reached (Fig. 1a). There was no difference between the growth curves for the microcarriers (Fig. 1a, b). The percent cells that attached to beads were also comparable between the beads (Fig. 1d). The distribution of cells across the microcarriers was different, however. Cytodex^®^ beads typically had more cells per bead then CellBIND^®^ and Synthemax^®^ II (Fig. 1e). Because of the better distribution of cells per bead for Cytodex^®^ we optimized the seeding density using these microcarriers.

**Fig. 2:**
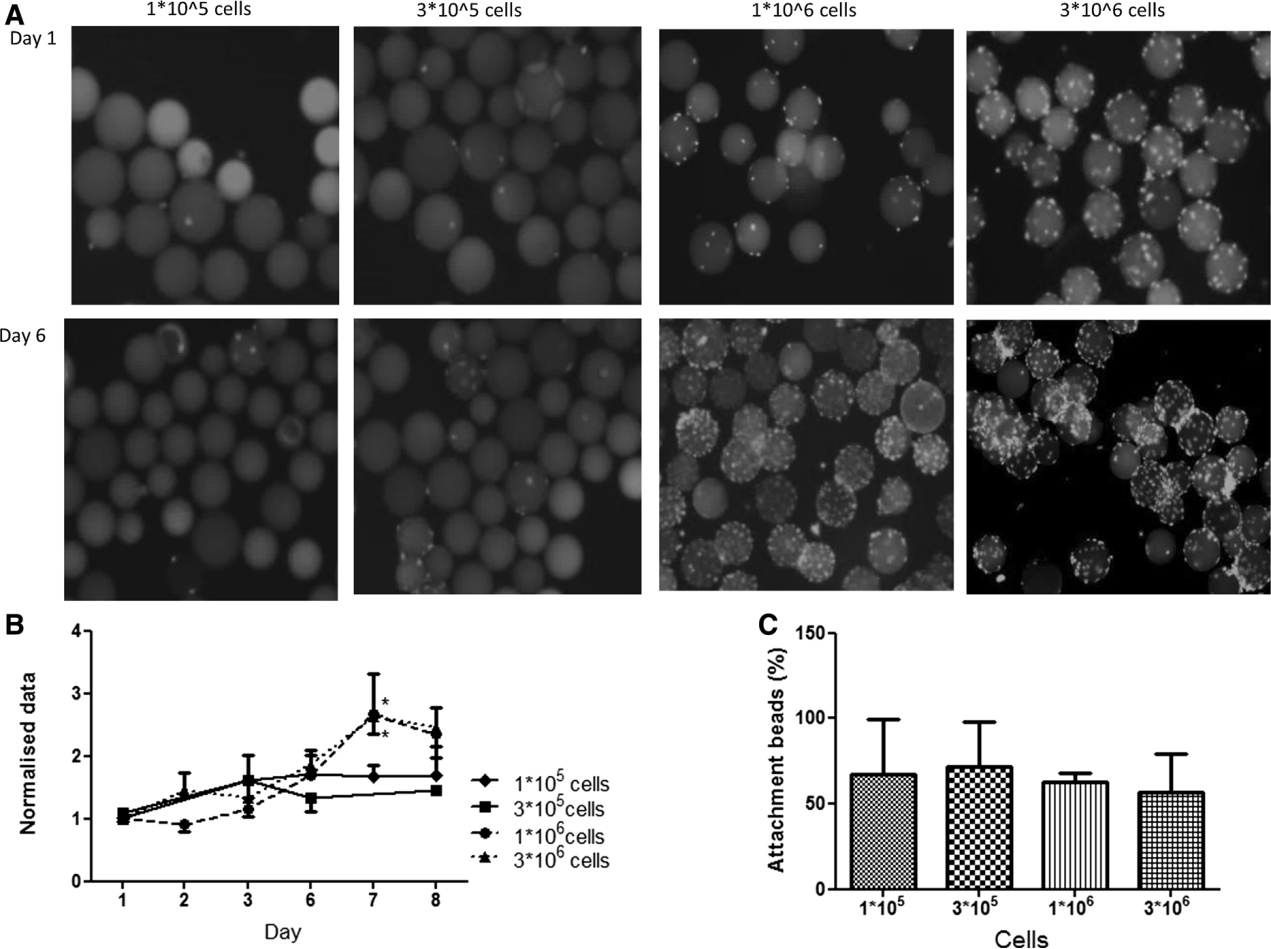
Myoblasts seeded on Cytodex^®^ 1 microcarriers with different seeding densities. **a** Photomicrographs of Cytodex^®^ 1 microcarriers with different seeding densities, at day 1 and day 6. **b** The *growth curve* of myoblasts with different seeding densities (n = 3/density/time point). DNA (μg/ml) was measured and normalised to the value at day 1. On day 7, cell numbers were significantly higher for densities of ≥10^6^ cells/condition. **c** Percentage attached cells to the beads after 24 h. *Asterisks* indicate *p* < 0.05

To determine the optimal seeding density, we made a concentration curve with 10^5^, 3 × 10^5^, 10^6^ and 3 × 10^6^ cells/ml on Cytodex^®^ 1 microcarriers in a spinner flask. Clearly, seeding densities below 10^6^ resulted in stationary cell numbers during the 6-day measurement (Fig. 2a, b). In contrast, the higher seeding densities showed proper exponential growth. At day 6, the highest seeding density of 3 × 10^6^ cells displayed aggregation of cells/microcarriers. Cell attachment to the Cytodex^®^ microcarriers was independent of seeding density (Fig. 2c).

### Bead-to-bead transfer

Transfer of cells from populated beads to empty beads would significantly reduce handling during expansion of the culture by just adding new beads to increase surface (Ferrari et al. 2014). Agitated stirring brings fresh beads in close contact with near confluent beads thus allowing cell transfer (Wang and Ouyang 1999).

**Fig. 3:**
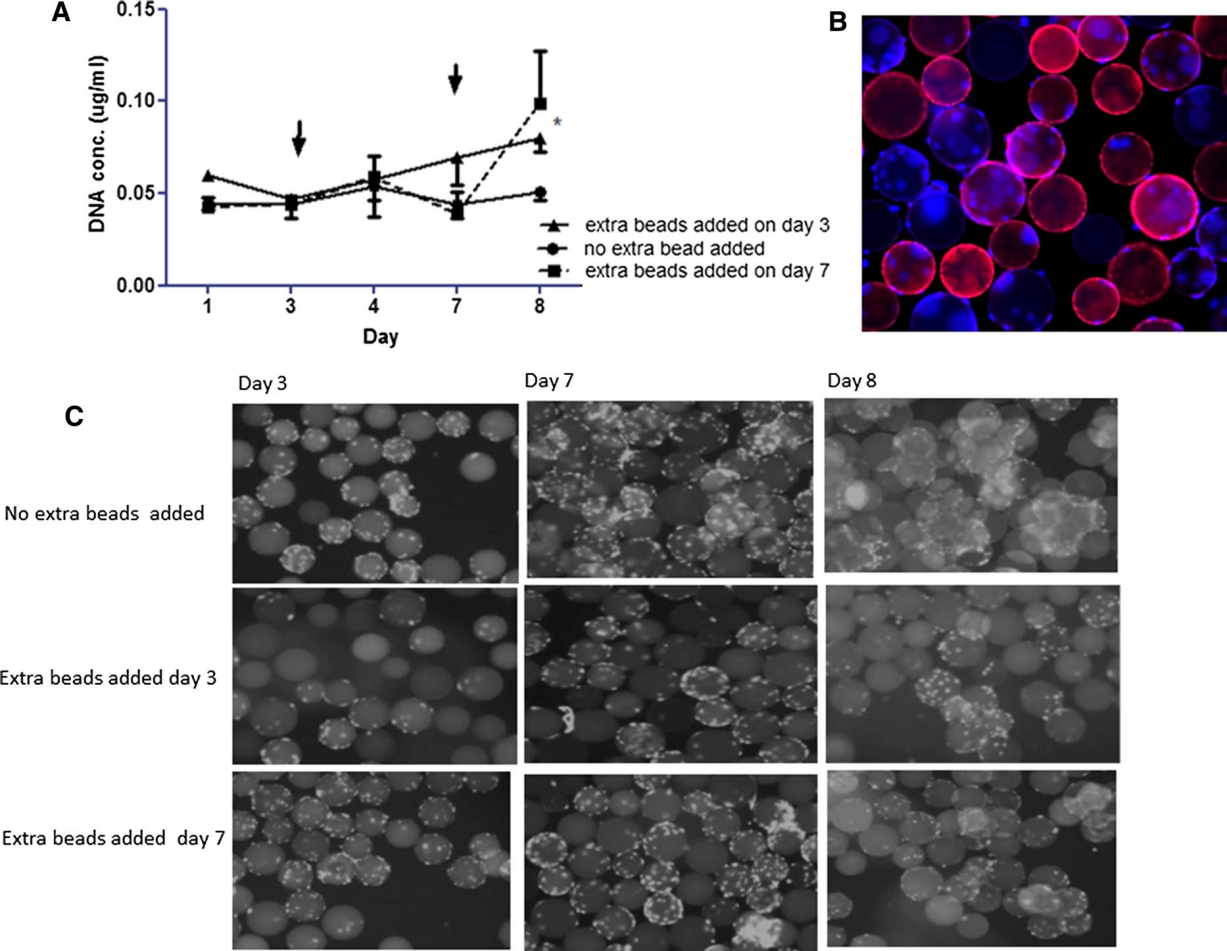
Bead-to-bead transfer of Myoblasts seeded on Synthemax^®^ II microcarriers. Synthemax^®^ II microcarriers were chosen for these experiments because they can be labeled with rhodamine. **a** The *growth curve* of the cells expressed as DNA concentration (μg/ml) where empty beads were added at day 3 or day 7. **b** Bead-to-bead transfer of myoblasts onto rhodamine (*red*) labeled Synthemax^®^ II beads. **c** Photomicrographs of the cells on beads at day 3, 7 and 8. *Asterisk* indicate significant difference for the growth when beads were added at day 7 compared to no extra beads added. (Color figure online)

Bead-to-bead transfer was tested using Synthemax^®^ II microcarriers as these can be stained with rhodamine. To an existing culture in stirrer flasks, new beads (5 ml 100 mg/ml beads) were added on day 3 and 7. Each bead addition was followed by 24 h of intermittent agitation. Growth was boosted by each bead addition (Fig. 3a) and transfer to newly added, rhodamine labelled beads was apparent (Fig. 3b). The addition of empty beads and subsequent bead-to-bead transfer also reduced the number of aggregates in comparison to cultures with no extra added beads (Fig. 3c, day 8).

### Myoblast differentiation

**Fig. 4:**
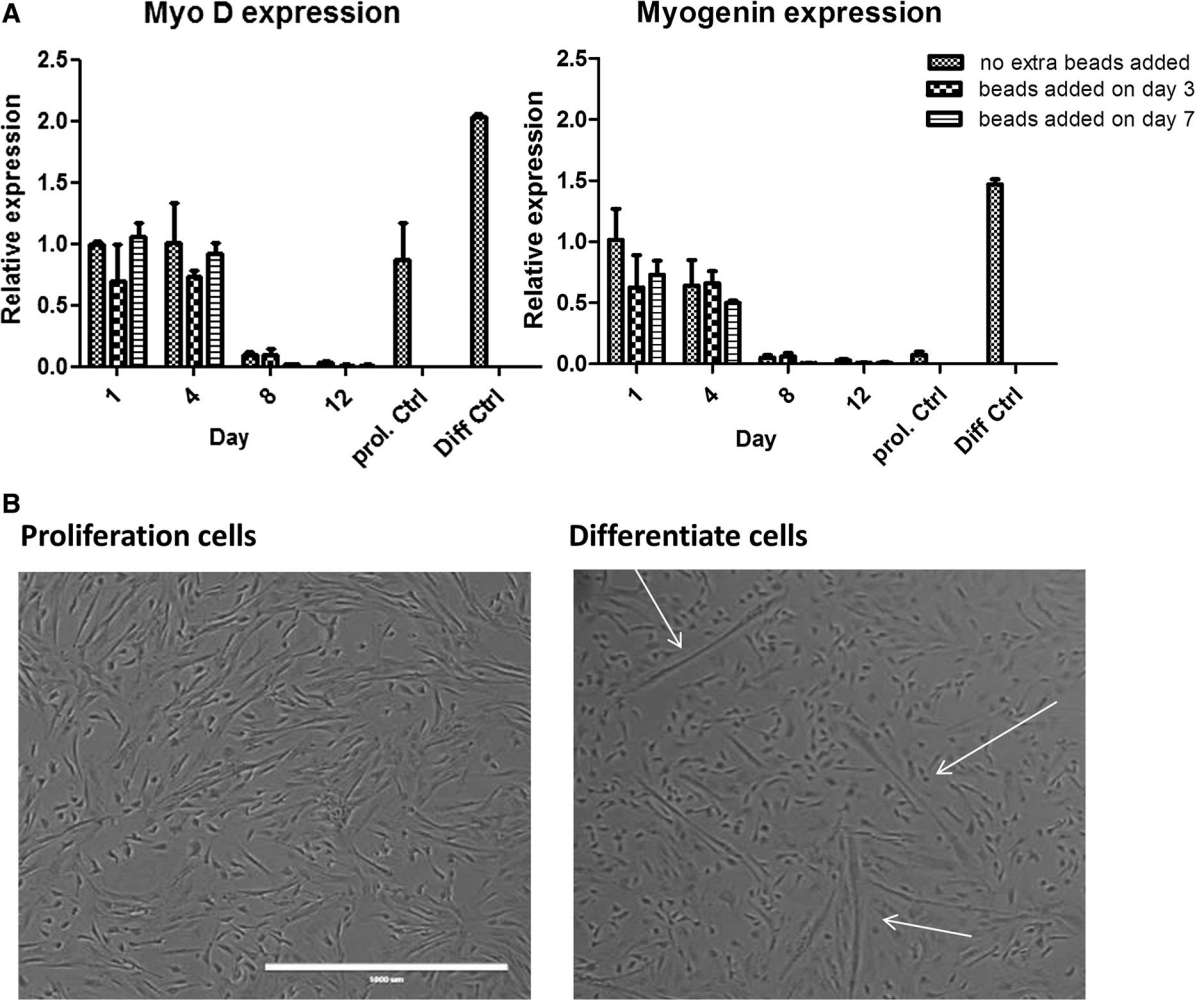
Myoblasts seeded on Cytodex^®^ 1 microcarriers, with new beads added on day 3 or day 7. **a** MyoD and Myogenin RNA expression by RT-QPCR. **b** The cells are trypsinized and then seeded on a plate in differentiation medium for 4 days to check myotube formation. Note the elongated structures that represent merged myoblasts. Scale bar = 1000 µm. “prol. Ctrl” is a 2D culture condition optimized for cell proliferation and “diff Ctrl” is optimized for myocyte differentiation. The *arrows* point to the myotubes

In 2D cultures reaching confluence, myoblasts show a tendency to differentiate into myotubes. As a measure of differentiation and capacity to differentiate after microcarrier-based cell expansion, expression of early differentiation markers MyoD and Myogenin was studied (Fig. 4a) as well as the appearance of myotubes (Fig. 4b). As expected, the expression of MyoD and Myogenin decreased over time during proliferation (Fig. 4a). This reduction could not be explained by changes in purity of the myoblast population, as this was constant and above 95% (data not shown). The cells retained their capacity to differentiate after they were transferred to 2D and differentiation medium.

## Discussion

In this study, we have established the proof of principle that bovine myoblasts can be cultured on microcarriers. No major differences were observed for the three tested microcarriers, except that the number of beads with low cell numbers was higher in CellBIND^®^ and Synthemax^®^ II beads suggesting a slower initiation of exponential growth. We therefore continued optimization with Cytodex^®^ 1. The optimal microcarrier may be cell specific. Goh et al. showed for human fetal mesenchymal stem cells that Cytodex^®^ 1, Cultispher^®^ and HyQSpheres resulted in higher cell numbers than Cytodex^®^ 3 (Goh et al. 2013). With a limited selection of microspheres but across different binding principles, we observed similar optimization settings as reported earlier for human mesenchymal stem cells and therefore did not confirm cell specificity.

Cell density optimization resulted in clearly better cell growth when the initial density was 1 × 10^6^ cells or higher (3 × 10^6^ cells). This is a common observation in cell culture, not only in microcarrier-based systems. It is assumed that paracrine factors of neighboring cells stimulate proliferation (Kimura et al. 1991). The optimal number of cells was 10^6^ cells/spinner flask (5500 cells/cm^2^ of bead surface). Higher densities resulted in early aggregate formation of cells and microcarriers. Aggregate formation depends on cell type i.e. the ability of cells to grow in multiple layers, and on the dynamic conditions of the culture (Muhitch et al. 2000). Early observations suggest that cell-microcarrier aggregate formation negatively affects growth of human mesenchymal stem cells (Chen et al. 2013; Caruso et al. 2014; Goh et al. 2013) and we assume that myoblasts behave similarly. Myoblasts seem to quickly participate in aggregate formation of microcarriers, suggesting that a tight schedule of adding new beads or starting a new passage will need to be followed for optimal cell growth.

The optimal initial cell density of 5500 cell/cm^2^ is comparable to earlier reports on mesenchymal stem cells in a microcarrier-based culture system (Hewitt et al. 2011; Rafiq et al. 2013). In 2D cultures, the optimal seeding density is typically lower (Coles et al. 2015), which is most likely illustrative of higher attachment to the static flat surfaces than to the spherical surface of highly dynamic microcarriers.

We provided direct evidence that bovine myoblasts display bead-to-bead transfer. Most experience on bead-to-bead transfer has been accumulated with mesenchymal stem cells (Ferrari et al. 2014). Myoblasts, similar to mesenchymal cells, reach confluence at day 3 or 4 after seeding, which is the optimal time to add new microcarriers. The two proposed mechanisms for bead-to-bead transfer are the exchange of cells during bead-to-bead contact or the pick-up of floating cells that have detached from confluent beads and are adopted by newly added, still barren, beads (Ferrari et al. 2014). The surprisingly sudden and profound increase in cell number that we observed after adding new beads can hardly be attributed to cell proliferation but could be compatible with the pickup of floating cells by the new beads. These detached cells would otherwise rapidly undergo anoikis as a result of inadequate cell–matrix interaction (Frisch and Screaton 2001), thus explaining the failure to display exponential growth in the absence of newly added beads.

Subculturing of cells is one of the critical risks to be considered in the scaling up of microcarrier-based cell culture as any manipulation can lead to contamination. Complete detachment of cells by way of trypsinization is challenging and incomplete detachment results in cell loss and production inefficiency (Caruso et al. 2014). Although bead-to-bead transfer may eliminate the need for subculturing, cells eventually need to be harvested while retaining their viability and propensity to differentiate. We have not quantitatively analyzed harvesting efficiency but we show here that the harvested cells are viable and capable of differentiation into myotubes.

As experiments were performed in spinner flasks with limited control over temperature, oxygen supply and nutrient availability and usage, the culture system may not be fully optimized for a large scale stirred tank bioreactor (Nienow 2006). Future studies in fully controlled stirred tank bioreactors need to result in further optimization.

The overall conclusion is that it is possible to culture bovine myoblasts on MC (Cytodex^®^ 1 or Synthemax^®^ MC) and that they exhibit bead-to-bead transfer. Bovine myoblasts seem to behave very similar to human mesenchymal stem cells. Thus, our results provide valuable data to further develop and scale up the production of bovine myoblasts as a prerequisite for efficient and cost-effective development of cultured meat. The similarity with microcarrier based culture of human mesenchymal stem cells, suggests that these results are also applicable to culture of anchorage dependent cells in medical tissue engineering and cell therapy.
